# Performance and Logistical Challenges of Alternative HIV-1 Virological Monitoring Options in a Clinical Setting of Harare, Zimbabwe

**DOI:** 10.1155/2014/102598

**Published:** 2014-06-15

**Authors:** Pascale Ondoa, Tinei Shamu, Michelle Bronze, Maureen Wellington, Tamara Sonia Boender, Corry Manting, Kim Steegen, Rudi Luethy, Tobias Rinke de Wit

**Affiliations:** ^1^Amsterdam Institute for Global Health and Development (AIGHD), Department of Global Health, Academic Medical Center, P.O. Box 22700, 1100 DE Amsterdam, The Netherlands; ^2^Newlands Clinic, 56 Enterprise Road, Newlands, Harare, Zimbabwe; ^3^Department of Molecular Medicine and Haematology, University of the Witwatersrand 7 York Road, Parktown, Johannesburg 2193, South Africa

## Abstract

We evaluated a low-cost virological failure assay (VFA) on plasma and dried blood spot (DBS) specimens from HIV-1 infected patients attending an HIV clinic in Harare. The results were compared to the performance of the ultrasensitive heat-denatured p24 assay (p24). The COBAS AmpliPrep/COBAS TaqMan HIV-1 test, version 2.0, served as the gold standard. Using a cutoff of 5,000 copies/mL, the plasma VFA had a sensitivity of 94.5% and specificity of 92.7% and was largely superior to the VFA on DBS (sensitivity = 61.9%; specificity = 99.0%) or to the p24 (sensitivity = 54.3%; specificity = 82.3%) when tested on 302 HIV treated and untreated patients. However, among the 202 long-term ART-exposed patients, the sensitivity of the VFA decreased to 72.7% and to 35.7% using a threshold of 5,000 and 1,000 RNA copies/mL, respectively. We show that the VFA (either on plasma or on DBS) and the p24 are not reliable to monitor long-term treated, HIV-1 infected patients. Moreover, achieving acceptable assay sensitivity using DBS proved technically difficult in a less-experienced laboratory. Importantly, the high level of virological suppression (93%) indicated that quality care focused on treatment adherence limits virological failure even when PCR-based viral load monitoring is not available.

## 1. Introduction

In 2011, 34 million people were estimated to be living with HIV/AIDS of whom 69% reside in sub-Saharan Africa [1]. Since the scale-up of antiretroviral therapy (ART), the number of African patients receiving ART has increased from 50,000 in 2003 to 7.5 million at the end of 2012 [1]. The number of HIV patients starting HIV treatment is expected to further increase with the global commitment aiming to provide ART to 15 million people by 2015 [2], as well as the gradual implementation of recent changes in WHO guidelines recommending earlier treatment initiation at 500 instead of 350 CD4 cell/mL [3]. In addition, the implementation of “treatment as prevention” will increase the number of HIV patients eligible for ART in low and middle income countries (LMIC) [4].

Despite the unquestionable success of internationally funded ART access programs, accumulating evidence suggests that sustaining patients on treatment and assuring quality of care is a formidable next challenge. In particular, the monitoring of the ART response based on the current clinicoimmunological parameters may be associated with prolonged virological failure, accumulation of HIV drug resistance (HIVDR) mutations, and inappropriate switching to second line treatment [5, 6]. Emergence of acquired HIVDR and subsequent onwards transmission of HIVDR reduces the predicted efficacy of ART regimens, increases the cost of HIV care, and therefore compromises the cost-effectiveness of ART programs in countries with limited resources.

Patient plasma HIV-1 viral load has been demonstrated to be the most sensitive and reliable marker of ART failure [7] and is now recommended by WHO as the preferred laboratory parameter to monitor response to ART whenever possible [3]. Based on recent evidence on the risk of HIV disease progression [8, 9], HIV transmission [10], and treatment failure [11], WHO has adjusted the threshold, defining virological failure, from 5,000 to 1,000 RNA copies/mL [3].

Despite this recommendation, the majority of clinical settings in sub-Saharan Africa cannot afford routine or even targeted VL monitoring because of the high price and complexity of current commercial PCR-based assays. The four FDA-approved viral load assays from Roche, Siemens, Abbott, and Biomérieux typically cost between US$ 40 and 125/test with prices varying as a function of the region, volume of samples, and negotiation with the supplier [12]. In addition, most of these VL tests are adapted to high-throughput testing and require dedicated laboratory space, expensive PCR equipment, experienced laboratory operators, and significant technical support and maintenance services. Hence, their implementation is generally not feasible in less-equipped and -experienced laboratories.

Several initiatives have been taken to provide alternatives for VL testing in resource limited settings. These include the measurement of indirect markers of viral replication such as the expression of the activation marker CD38 on CD8 lymphocytes by flow cytometry [13, 14], the measurement of heat-denatured HIV-1 core protein p24 [15, 16] or HIV-1 reverse transcriptase activity [17–19] in plasma. Another approach has been the development of simpler, cheaper assays for HIV-1 viral load determination, based on currently available technologies, for example, the LTR-based open platform PCR assay from Biocentric [20–22]. To date, implementation of these alternative viral load assays remains limited in resource constrained settings due to their cost (e.g., US$ 30 for ExaVir Load and US$ 20 for the Generic HIV-1 viral load from Biocentric) [12], absence of FDA-approval, lack of comparability with existing VL gold standards, and so forth. In addition, the use of some of these assays is currently not supported by WHO, due to the lack of sensitivity and/or specificity to detect virological failure, as compared to existing gold standard assays (this is the case for p24 and ExaVir Load assays).

In light of the above, our team developed the Affordable Resistance Test for Africa (ARTA) virological failure assay (VFA): http://www.arta-africa.org/. The assay is designed as an open platform and is based on real-time PCR of the HIV-1 LTR fragment as described elsewhere [23, 24]. The VFA is HIV-1 subtype independent, applicable to lower throughput settings and can be applied to dried blood spots (DBS), thanks to the optimized nucleic acid elution methods. The VFA is a qualitative assay that classifies samples around a single predefined Ct-value into positive (virological failure) and negative (nonvirological failure) results.

So far, the VFA has been evaluated on purposefully selected panels of HIV-1 infected plasma and in laboratory-controlled conditions [23, 24]. In order to further demonstrate its clinical utility, we have set out to evaluate the VFA in a clinical setting, using consecutive clinical samples from patients attending Newlands Clinic, an HIV treatment center in Harare, Zimbabwe. This site was chosen because of its good reputation in terms of ART clinical management, its experience with another alternative VL assay: the heat-denatured p24 test [25] and its long-term involvement in ART patient cohort follow-up, including HIV drug resistance monitoring [26]. At Newlands Clinic, we compared the performance of the newly implemented VFA to the routinely used heat-denatured p24 assay (p24), using the results of the COBAS AmpliPrep/COBAS TaqMan HIV-1 test, version 2.0 (VL_ref_), performed in the reference laboratory, as the gold standard. All three assays were performed on clinical samples from ART naive and long-term treated HIV-1 infected patients. The group of treated patients is especially relevant in terms of assessing to what extent the VFA and the p24 can reliably identify true virological failures and contribute to the adequate management of ART.

Performance outcomes, benefits, and operational challenges of the alternative assays in this clinical setting are discussed.

## 2. Materials and Methods 

### 2.1. Study Site

Newlands Clinic is a family centered, nurse based HIV care and treatment center based in Harare, Zimbabwe, which was founded in 2004. It is a part of the coordinated public-private partnership between the Ministry of Health and Child Care and provides access to care and treatment to over 4000 HIV-1 infected patients from marginalized communities within urban and periurban Harare and Chitungwiza. Patient care follows the national HIV treatment guidelines of Zimbabwe [27]. Patients are typically seen once every month, but those that are stable on antiretroviral therapy with demonstrated good adherence are seen once every three months. The staff compliment includes 17 nurses who play the leading role in patient care and two doctors, respectively, supporting the adult, and the pediatric and adolescent patient management. The clinic also houses a laboratory and a pharmacy which provide quick turnaround of laboratory investigations and convenient drug pick-up after consultations. These services are provided at no cost to the patients. The laboratory did not have access to PCR-based HIV-1 viral load measurement at the time of study. Since 2004 and up to the study completion, Newlands Clinic patients receiving ART were monitored using the ultrasensitive p24 assay [15]. The results from the p24 assay were used by the clinical staff to identify patients more likely to experience virological failure and to benefit from adherence counseling, according to an in-house protocol.

### 2.2. Patients and Samples

Matched plasma and DBS were collected at single time points from 202 HIV-1 infected patients participating in a long-term observational cohort study [28] and who were reporting for their month 36 visit after the initiation of 1st or 2nd line ART. In addition, 100 HIV-1 infected patients not (yet) eligible for ART, attending Newlands Clinic for routine care were included. Ethical approval for the study was obtained from the Research Council of Zimbabwe (RCZ) and the Medical Research Council of Zimbabwe (MRCZ). Written informed consent was obtained from all study participants. Demographic information and clinical and laboratory data were collected according to the clinic algorithm at each visit and recorded in an electronic medical record system.

### 2.3. Sample Preparation and Storage

Seven mL of EDTA blood was drawn from all patients. Fifty *μ*L of blood was immediately used for CD4 count determination and the preparation of two DBS cards containing five spots of 50 *μ*L each. Plasma was separated within 2 hours after phlebotomy. Each plasma sample was aliquoted into six cryovials and stored at −80°C until further testing. DBS were stored at −20°C in plastic bags containing desiccant until further use.

For each patient, two aliquots of plasma were shipped on dry ice to the reference laboratory of Witwatersrand University, Johannesburg, South Africa, where reference VL determination was performed (see the following), according to international quality standards and procedures. The remaining plasma aliquots were kept on site for VFA and p24 testing (see the following).

### 2.4. Laboratory Tests

#### 2.4.1. Ultrasensitive Heat-Denatured p24 Antigen

p24 antigen concentration was measured on all plasma specimens using the Ultrasensitive p24 Ag ELISA kit (Perkin-Elmer Life Sciences, Boston, MA), following the procedure previously described [15].

The optimal cutoff to define virological failure was determined using the ROC curve (see the following).

#### 2.4.2. Reference Viral Load

VL_ref_ was measured using the COBAS AmpliPrep/COBAS TaqMan HIV-1 test, version 2.0 (Roche Molecular Diagnostic Systems, Branchburg, NJ), as per manufacturer's instructions. The assay has a detection limit of 20 RNA copies/mL.

#### 2.4.3. ARTA VFA

The VFA is based on real-time PCR targeting the long terminal repeat domain (LTR) of HIV-1 [24] and was designed to be used as a qualitative viral load test. Quality of testing is controlled at every step by the incorporation of an internal control (IC) in each clinical sample. The IC comprised of the nonhuman RNA virus, encephalomyocarditis virus (EMC), prepared at the UMCU, Utrecht, The Netherlands. Nucleic acids from plasma were extracted using the NucliSENS easyMAG System (bioMérieux) as per manufacturer's instruction, with an on-board lysis incubation as previously described [23, 24].

Nucleic acid extractions from DBS were performed using an initial off-board lysis step, using two DBS (estimated 50 *μ*L whole blood/plasma per spot) and incubating them in the NucliSENS Lysis Buffer (2 mL) for an hour at room temperature. The DBS paper was removed from the lysis buffer, and five *μ*L of the internal control was spiked into each lysate. Two ml lysate was aliquoted into a NucliSENS easyMAG sample vessel and eluted in 25 *μ*L of elution buffer. The downstream extraction process was conducted in the same manner as for the plasma samples.

Previous evaluations [24] have determined the levels of detection for clinical samples (LOD, i.e., the lower viral load concentration where no negative VFA results were observed) to be 1,000 RNA copies/mL for plasma and 5,000 RNA copies/mL for DBS. The classification of results into virological or nonvirological failures is done by using the site-specific threshold Ct-value corresponding to either 5,000 copies/mL (for both plasma and DBS) or 1,000 copies per mL (for plasma only).

Three aliquots from a well-characterized plasma specimen, with VL > 7Log_10_ RNA copies/mL, were obtained from UMCU in Utrecht and were used to construct a standard curve. One in ten serial dilutions ranging from 7Log_10_ to 2Log_10_ RNA copies/mL of each plasma aliquot was tested with the VFA to generate a standard curve. Mean ± 2 standard deviations (SD) of the Ct-values corresponding to 5,000 and 1,000 RNA copies/mL were calculated from the standard curve and were used as thresholds to categorize samples into virological or nonvirological failure. For the purpose of analyzing qualitative data, Ct-values translating down to 250 RNA copies in the plasma and 1,000 copies in the DBS specimen were reported. These values represent the lower VL values at which the respective assays keep their linearity.

Viral load determination of clinical samples was performed as previously described [23, 24] using a MiniOpticon Real-Time PCR system (Life Technologies, CA, USA) and the analysis was done using the MiniOpticon software. All Ct-values < mean + 2SD were categorized as virological failure. All Ct-values > mean − 2SD were reported as negative. Ct-values falling within mean ± 2SD were retested and, if still within this range, classified as virological failure.

### 2.5. Assay Costing

The calculation of the VFA cost included the price of the equipment, reagents (including shipment), consumables, and labour. The cost of the VFA in Zimbabwe was compared to the cost of the VFA as performed in the reference laboratory in South Africa.

### 2.6. Data Analysis

In treated patients, immunological failure was defined as CD4 count measured at month 36: (1) equal or lower than baseline values; (2) lower than 100 cells/uL; or (3) lower than 50% of treatment peak value (measured at month 12 or month 24), according to the WHO guidelines for ART monitoring [3].

Undetectable viral load results were given a value representing the average between 0 and the lower detection limit for each test: VL_ref_ = 10 copies/mL, VFA on plasma = 125 copies/mL, and VFA on DBS = 500 copies/mL. VL_ref_, VFA, and p24 results were log-transformed prior to quantitative analysis to approach normal distribution.

Data analysis was performed using both the previous (5,000 RNA copies/mL) and current (1,000 RNA copies/mL) cutoff values to define virological failure according to WHO guidelines [3]. Individual assay performance in detecting virological failure was determined by calculating the percentage of correctly classified samples (virological failure or nonvirological failure), sensitivities, and specificities, using the VL_ref_ as the gold standard. Sensitivity was defined as the number of samples testing positive, using the assay being evaluated, reported to the total number of true virological failures as per VL_ref_. Specificity was defined as the number of samples testing negative with the assay being evaluated, reported to the total number of true nonvirological failures as per VL_ref_. Comparison between assay performances was determined using area under the ROC curve (AUC). The Youden index: max[(sensitivity + specificity)−1] was used to define the optimal p24 cutoff value to determine virological failure at 5,000 or 1,000 RNA copies/mL as per VL_ref_ assay. Differences between groups were calculated using the Student's* t*-test for numerical values and the chi-square test for categorical data. The level of significance of *α* was set at 0.05. Agreement between quantitative results of VFA, the p24, and VL_ref_ was evaluated using a Bland Altman analysis.

Data were analyzed using SPSS version 20.0 (IBM Corporation, Armonk, NY) and Graph Pad Prism version 6.00 for Windows (GraphPad Software, San Diego CA).

## 3. Results 

### 3.1. Characteristics of the Study Population

One hundred ART-naive and 202 ART patients treated for at least 36 months were included in the study. There were no significant differences in age and gender between the two patient groups (Table 1). Most patients were women; all viruses from ART-exposed patients belonged to HIV-1 subtype C, as described elsewhere [26]. Viral subtype was not available for ART-naive patients. Hemoglobin levels were marginally lower in ART-naive patients (Table 1). Treated patients were significantly less represented in the lowest CD4 category (≤200/*μ*L) as compared to the ART-naive group (17.1% versus 9%). Thirty-eight patients (18.8%) of the treatment-exposed group had immunological failure.

Among the 100 ART-naive patients, 81 had a VL_ref_ > 5,000 copies/mL and 92 a VL_ref_ > 1,000 copies/mL. Among the ART-treated patients these figures were 11 (5.4%) and 14 (6.9%), respectively.

### 3.2. VFA Evaluation 

#### 3.2.1. Determination of VFA Cutoff Values for the Identification of Virological Failure

The standard curve was built from testing serial dilution of one well-characterized plasma aliquots (data not shown). Mean Ct-values and SD corresponding to 5,000 and 1,000 RNA copies/mL were extrapolated from the equations of three individual standard curves. Thresholds Ct-values defined as [mean ± 2SD] were calculated and equaled to 36.65 ± 0.04 for a cutoff of 5,000 copies/mL and 38.56 ± 0.35 for a cutoff of 1,000 copies/mL. The same threshold Ct-values were used for both plasma and DBS.

#### 3.2.2. Diagnostic Capacity of the VFA in Plasma and DBS for the Identification of Virological Failures

A total of 302 specimens had VL_ref_ results. Three hundred one plasma specimens were tested with the p24 assay and 300 with the VFA on plasma. Two hundred ninety-nine DBS specimens were tested with the VFA.

Area Under the ROC Curve (AUC, data not shown) indicated that the capacity to predict virological failure as per VL_ref_ was the highest in the plasma VFA (AUC = 0.980 and 0.981 when using a threshold of, resp., 5,000 or 1,000 RNA copies/mL as per VL_ref_) followed by the DBS VFA (AUC = 0.910, using the threshold of 5,000 RNA copies/mL only).

Overall, the percentage of samples correctly classified was the highest with plasma VFA, (93.3% using 5,000 RNA copies/mL and 93.0% using 1,000 RNA copies/mL, as thresholds, Table 2), whereas the VFA on DBS correctly classified 87.6% of the samples, using a threshold of 5,000 RNA copies/mL. The percentage of correctly classified samples using the VFA in plasma was irrespective of the threshold regardless whether patients were treated or not. However, a dramatic decrease of VFA sensitivity was observed for both plasma and DBS when only treated patients were considered (e.g., sensitivity of plasma VFA = 94.5% among all patients versus 72.7% among treated patients, when using a cutoff of 5,000 RNA copies/mL, Table 2). In the group of treated patients, only one of 11 virological failures could be detected based on the results of the VFA DBS (sensitivity = 9.0%, Table 2), highlighting possible issues with the application of DBS in this setting.

#### 3.2.3. Diagnostic Capacity of the p24 Assay to Identify Virological Failures

The AUC of the p24 assay was 0.714 using the threshold of 1,000 RNA copies/mL and 0.715 using the threshold of 5,000 copies/mL, reflecting the lower diagnostic capacity of this assay as compared to the VFA (data not shown). The Youden index indicated that the optimal cutoff to identify virological failure using the p24 assay was 3 pg/mL for the threshold of 5,000 RNA copies/mL and 2.65 pg/mL for the threshold of 1,000 RNA copies/mL as per VL_ref_. These two cutoff values were used for the calculation of sensitivities and specificities. The p24 assay demonstrated a poor capacity to identify VL_ref_ > 1,000 RNA copies/mL (sensitivity = 54.3%) or VL_ref_ > 5,000 copies/mL (sensitivity = 49.0%) with more than a quarter of the samples being misclassified using either threshold (Table 2). Sensitivities and specificities of the p24 assay were similar in the group of treated as compared to ART-naive patients (Table 2).

#### 3.2.4. Agreement between Plasma VFA and VL_ref_

**Figure 1:**
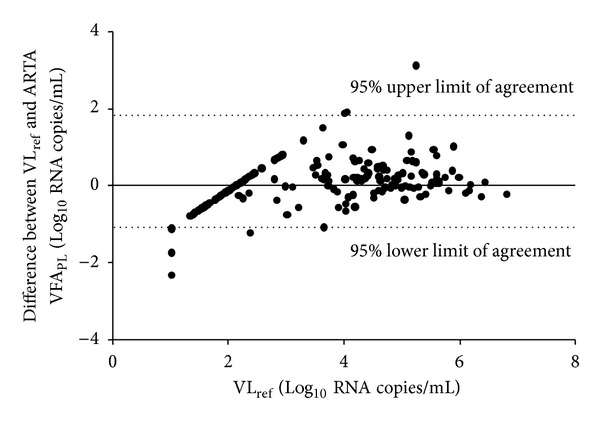
Bland-Altman analysis of plasma using the VL_ref_ as the gold standard.

Although the VFA is not designed for quantitative VL determination, we conducted a Bland Altman analysis to further explore the agreement between measurements with plasma VFA and the VL_ref_ (Figure 1). The graph shows mean Log_10_ differences between VL_ref_ and plasma VFA plotted against VL_ref_Log_10_ values. The analysis of the plasma VFA against VL_ref_ revealed a bias of −0.384 with an SD of 0.743, indicating that the VFA on plasma tends to slightly overestimate the VL. Overall, the assay had the tendency to better correlate for samples with VL_ref_ above 4Log_10_ RNA copies/mL. This was illustrated by most of the false negative results occurring with samples having VL_ref_ ≤ 4Log_10_ RNA copies/mL. One outlier specimen with VL_ref_ of 5.3Log_10_ RNA copies/mL, however, was not detected by the plasma VFA. Retesting of this sample with the VL_ref_ confirmed viral load >5Log_10_ RNA copies/mL (data not shown). Additional disagreements were observed with 4 samples giving false positive values using plasma VFA as compared to VL_ref_.

#### 3.2.5. Characteristics of Misclassified Samples Using VFA on Plasma from Treated and ART-Naive Patients

Using a threshold of 5,000 RNA copies/mL, the plasma VFA misclassified 5 samples as false negative and 15 samples as false positive (see Table 2(a)). Using a threshold of 1,000 RNA copies/mL, the plasma VFA misclassified 21 samples as false negative, with no false positive samples (see Table 2(b)). The comparison of correctly (true positive) versus misclassified (false negative) virological failures (*n* = 106), using a threshold of 1,000 RNA copies/mL, indicated an overrepresentation of treated patients amongst the false negative (9 treated patients of a total of 21 misclassified versus 5 treated patients of total 85, correctly classified, *P* < 0.0001, Table 3). This higher proportion of specimen from treated patients among under-called samples was accompanied by significantly lower VL_ref_ (mean VL_ref_ = 3.82Log_10_ RNA copies/mL among the misclassified patients versus 4.9Log_10_ RNA copies/mL among the correctly classified patients, *P* < 0.0001). No differences in gender, age, or CD4 count were observed between correctly classified and misclassified virological failures. Findings were similar when the analysis was done using a threshold of 5,000 RNA copies/mL to define virological failure (data not shown).

Conversely, among a total of 210 samples with VL_ref_ ≤ 5,000 RNA copies/mL, VL_ref_ values were significantly higher in the 15 samples classified as false positive using the plasma VFA as compared to the 195 correctly classified samples (true negative, VL_ref_ = 3.14Log_10_ RNA copies/mL versus VL_ref_ = 1.4Log_10_ RNA copies/mL, *P* < 0.0001, data not shown).

#### 3.2.6. VL_ref_, VFA, and p24 Levels in Groups of Treated Patients with Different Immunological Outcomes

In order to explore the utility of the three assays beyond the identification of virological failures, we compared levels of viral load as per VL_ref_ and plasma VFA as well as levels of p24 concentration between groups of patients categorized as a function of WHO-defined immunological failure. Both VL_ref_ and plasma VFA quantitative measurements were significantly higher in patients with immunological failure (*P* < 0.0001 and *P* = 0.001, resp., data not shown) as compared to immunological responders. In contrast, there was no significant difference in p24 concentration in immunologically failing patients as compared to the other participants (*P* = 0.882, data not shown).

#### 3.2.7. VFA Costs

The cost of the VFA in plasma was calculated based on one run of 19 plasma samples, including positive and negative controls. Labor costs were based on an average of 2.5 hours spent by one laboratory technician per test run. Overall, the cost of the VFA in Zimbabwe (US$ 30.31) was very similar to South Africa (US$ 28.5, Table 4) The labour cost/sample was increased in Zimbabwe due to the platform used, which only allowed for 19 specimen to be tested/run, as opposed to the instrument used in South Africa, which allows for 926 samples to be processed concurrently. Fixed instrument expense was also higher in Zimbabwe.

## 4. Discussion

This study evaluated the performance of an alternative, open-platform, low-cost VL assay (VFA) in plasma and DBS as compared to the p24 assay, using the commercial viral load assay from Roche (CAP/CTM) as the gold standard for the identification of virological failure in a group of treated and ART-naive HIV-1 infected patients. The evaluation was done in a local setting and included samples from patients treated for at least 36 months, in whom the performance of an alternative viral load assay is most relevant to study.

The data indicate that the plasma VFA showed the best performance in identifying virological failures as compared to the DBS VFA and the p24 assay, using a cutoff of either 5,000 or 1,000 RNA copies/mL as per VL_ref_. AUC, sensitivities, and specificities of the plasma VFA were comparable to previous reports on similar panels of samples [23, 24]. In contrast, the performance of the VFA on DBS was lower than expected, with a higher proportion of under-called samples resulting in a poor sensitivity (61.9% compared to more than 90% in previous evaluations) [23, 24]. The underperformance of the VFA on DBS, whilst plasma samples gave substantially better results, suggests possible issues with (long-term) storage of the specimens or to the lack of experience of the operator. DBS are an easy-to-collect sample type allowing storage at room temperature, which is a real advantage for resource-low setting [29]. However, it has been demonstrated that the stability of RNA may be compromised of conditions such as high temperature or humidity [30, 31]. In addition, nucleic acid extraction from DBS starts with lower volume of blood per spot as compared to liquid plasma and is known to be cumbersome. Hence, the need of sufficient proficiency in order to achieve adequate rates of nucleic acid amplification from DBS specimen, especially in samples with lower viral load [24, Aitken et al., 2014, unpublished]. This report suggests that despite the encouraging results of optimized nucleic acid extraction from DBS in laboratory-controlled conditions, further improvement and standardization of DBS-based assay protocols are needed to allow their application for the monitoring of long-term treated patients in less-experienced laboratory settings. As previously observed [23, 24], the utilization of an extraction method isolating both DNA and RNA did not appear to affect the specificity of the DBS assay.

Overall, the p24 assay had a poor capacity to classify samples into virological or nonvirological failure, regardless of the cutoff used. More than a quarter of the samples tested were misclassified mostly due to overcalling of the VL_ref_ (false positive). Despite initial observations indicating that heat-denatured p24 is a good alternative to HIV-1 RNA load [15], subsequent assessments of the p24 utility for patient monitoring have produced mixed results [16]. Several studies have reported the poor correlation between p24 and RNA VL or CD4 changes during ART [32, 33]. Our finding is in line with the previously reported lack of kinetic synchronicity between HIV-1 p24 and RNA [34, 35]* in vivo*. This translates into p24 reactivity being measured in samples from patients receiving long-term ART where HIV-1 RNA is undetectable [25], presumably related to the p24 assay detecting p24 molecule present outside the viral particles. Conversely, under-detection of p24 in samples with measurable RNA viral load may be due to the insufficient or reversible disruption of p24 immune complexes in the plasma during the heat-denaturation process.

The sensitivity of VFA in plasma and DBS decreased substantially in the group of long-term treated patients. A plausible explanation is that VL associated with virological failure might be relatively lower than those measured in ARV-naive individuals, with greater odds of being classified as false positives by the VFA. Previous evaluations of the VFA were conducted on panels of samples artificially composed to cover very high to low levels of viral load, regardless of ART exposure [23, 24]. Our study shows that sensitivities and specificities calculated in this context may not reflect the clinical reality of monitoring long-term HIV-1 treated patients, who are mostly virologically suppressed. Future evaluations of similar assays should include more samples with VL levels in the vicinity of the cutoff values.

The present findings do not support the use of the VFA (either on plasma or on DBS) or the p24 for the reliable monitoring of long-term virological response to ART. To date, no consensus has emerged on acceptable rates of misclassification for the field use of alternative VL assays. However, it is our feeling that missing more than 20% of patients failing their treatment is unacceptable, given the fact that false positive patients are not eligible for a retesting before the next (bi-)annual visit [3], in the absence of clinical signs. Despite relatively low rates of virological failure in cohorts of long-term treated patients, the risk of emerging or accumulating HIV drug resistance mutations is potentially high in undiagnosed virological failure.

Interestingly and in contrast with VL_ref_ and VFA, there was no association between higher p24 levels and more advanced disease stages as defined by CD4 count. Although intrapatient longitudinal changes of p24 concentration have been shown to have some value in identifying patients at risk of disease progression [36, 37], our findings collectively suggest that cross-sectional measurements of p24 do not contribute significantly to the reliable and timely identification of patients with virological failure. Based on other observations, Newlands Clinic recently dropped the p24 assay and switched to a commercial PCR-based VL technology to monitor ART.

Our observations indicate that the VFA technology transfer to a less-sophisticated laboratory was feasible in this setting. Procurement of reagents and consumables is one of the main barriers limiting adequate operation of medical laboratories in sub-Saharan Africa. Hence, careful consideration and planning are needed when implementing a new assay in the field; especially when this assay is based on an open platform. In this case, instruments and reagents were procured relatively fast and at a reasonable price, albeit with significant logistical and administrative assistance from the reference laboratory in South Africa. Importantly, this report indicates that the price of US$ 30.31/test is only 12% cheaper than the discounted NucliSENS EasyQ HIV-1 v1.2 previously reported for a reference laboratory in South Africa [23]. Irrespective of the VFA performance, such a price may remain prohibitive for the majority of resource limited settings. However, with most of clinical sites not in the position to negotiate wholesale prices from reagent supplier, the cost of assays similar to the VFA may still allow savings up to US$ 10 to 90/test [16, 38].

The duration of the training was brief (two weeks), since the operator had received previous training in molecular biology techniques, which significantly alleviated the learning curve. At the end of the training, it took 2.5 hours for the laboratory technician to process one test run of 19 samples, which is the average time required for this type of assay.

The high level of viral suppression rate at month 36 on treatment of PASER patients (93%) was achieved in the absence of any sensitive virological monitoring and underscores the excellent clinical practice provided by the site. In addition, the high percentage of patient retention (82%) and the previously reported low level of acquired HIV-DR at month 12 [39] demonstrate that although VL is an irreplaceable tool for monitoring virological response, the clinical care focused on patient support provided at Newlands Clinic is central to the achievement of several WHO-suggested targets of HIV drug resistance prevention [40, 41].

This report demonstrates the feasibility of implementing a PCR-based VL assay in a less-experienced, low throughput laboratory, with the plasma VFA results comparable to those obtained in accredited reference laboratory in South Africa and in The Netherlands. Adequate performance of the VFA on DBS was however not achieved in this setting, indicating that using this type of specimen for molecular assays requires more technical proficiency as compared to liquid plasma.

This study indicates that neither the VFA nor the p24 are reliable tools to identify virological failure to ART in long-term treated patients, who are mostly virologically suppressed. The routine use of affordable VL testing is largely advocated to preserve the cost-efficiency of ART programmes in resource limited settings [42, 43]. It may be argued that a poorly sensitive VL load assay may still surpass clinicoimmunological criteria for the detection of virological failure. However, the implementation of VL monitoring technology requires significant investment. In order to avoid the fact that precious resources are not diverted away from scaling up the access to ART, it is hence of crucial importance that VL assays to be rolled out are carefully chosen to reach sufficient sensitivity using a threshold of 1,000 RNA copies/mL in the context of low prevalence of virological failure. Based on these observations, further development and evaluation of the VFA have been stopped by the ARTA consortium and the use of the p24 has been replaced by a commercial VL assay at Newlands Clinic.

The good clinical outcome of treated patients at this site suggests that despite the absence of conventional virological monitoring, quality clinical care, focused on patient support for treatment adherence, contributes to limit virological failure, thereby preventing the emergence of HIV drug resistance.

## Figures and Tables

**Table 1:** Characteristics of the study population.

	ART-naive (*n* = 100)	ART-treated (*n* = 202)	*P*
Age (years)			NS
18–29	19 (19)	12 (5.9)^¥^	
30–49	74 (74)	153 (75.7)	
>50	7 (7)	35 (17.3)	
Gender			NS
Male	32 (32)	72 (35.6)	
Female	68 (68)	130 (64.3)	
HIV-1 subtypes	Not available	all subtype C	
Hemoglobin (g/dL, mean, [min, max])	12.4 [5.9–17.30]	12.9 [7.40–16.9]∗	0.055 (*t*-test)
CD4 count (cells/mL) *N* (%)			*P* = 0.02 (Pearson chi-square)
≤200 cells/mL	17 (17.1)	18 (9.0)^§^	
251–350 cells/mL	23 (23.2)	58 (29.2)	
351–500 cells/mL	28 (28.2)	62 (31.3)	
>500 cells/mL	31 (31.3)	56 (28.3)	
Immunological failure	Not applicable	38 (18.8)	
Positive VL/virological failure			
Ref VL > 5000 cp/mL	81 (81)	11 (5.4)	*P* < 0.000 (Pearson chi-square)
Ref VL > 1000 cp/mL	92 (92)	14 (6.9)	*P* < 0.000 (Pearson chi-square)
Positive p24 Ag (VL_p24_ > 3 pg/mL)	51 (51)	36 (17.8)∗	*P* < 0.000 (Pearson chi-square)

^§^ *n* = 194, *n = 201, and ^¥^ *n* = 200.

**Table:** (a) All patients (*n* = 302).

	Number tested	Correctly classified	Misclassified	Undercalled	Overcalled	Sensitivity	Specificity
VFA plasma (CO = 5,000 cp/mL)	300	280 (93.3%)	20 (6.6%)	5/20	15/20	94.5%	92.7%
VFA plasma (CO = 1,000 cp/mL)	300	279 (93.0%)	21 (7%)	21/21	0/21	80.1%	100%
VFA DBS (CO = 5,000 cp/mL)	299	262 (87.6%)	37 (12.3%)	35/37	2/37	61.9%	99.0
p24 (CO = 5,000 cp/mL)	301	222 (73.5%)	79 (26.2%)	42/79	37/79	54.3%	82.3%
p24 (CO = 1,000 cp/mL)	301	216 (71.7%)	85 (28.2%)	52/85	33/85	49.0%	83.0%

**Table:** (b) Treated patients (*n* = 200).

	Number tested	Correctly classified	Misclassified	Undercalled	Overcalled	Sensitivity	Specificity
VFA plasma (CO = 5,000 cp/mL)	200	194 (97.0%)	6 (3.0%)	3/6	3/6	72.7%	98.0%
VFA plasma (CO = 1,000 cp/mL)	200	191 (95.5%)	9 (4.5%)	9/9	0/9	35.7%	100%
VFA DBS (CO = 5,000 cp/mL)	199	189 (95.0%)	10 (5.0%)	10/10	0/10	9.0%	100%
p24 (CO = 5,000 cp/mL)	201	166 (82.5%)	35 (17.5%)	5/35	30/35	54.7%	84.2%
p24 (CO = 1,000 cp/mL)	201	163 (81.0%)	48 (19.0%)	8/38	30/38	42.8%	83.9%

**Table 3:** Characteristics of correctly classified versus misclassified virological failures as per the VL_ref_ and using a cut-off of 1000 copies/mL (*N* = 106).

	Correctly classified *N* = 85	Misclassified *N* = 21	*P*
Treated patients	5 (5.8%)	9 (42.8%)	0.0001^*¥*^
ART-naive	80 (94.1%)	12 (57.1%)	
Male/female ratio	0.63	0.50	0.642^¥^
Age (years)	36.5	37.8	0.590^*£*^
CD4 count (mean cells/uL)∗	364	411	0.367^*£*^
Hb (mean g/dL)∗∗	12.43	12.76	0.557^*£*^
VL_ref_ (mean log_10_⁡RNA copies/mL)	4.90	3.82	0.0001^*£*^

*n = 104, **n = 105, ^¥^chi-square, and ^£^ *t*-test.

**Table 4:** Assay costing.

Cost per samples	Wits laboratory (based on 92 samples)	Newlands clinic (based on 19 samples)
Labour cost	0.2	1.34
Fixed instrument expense	0.3	0.74
Reagent + consumable cost	28	28.23
Total cost	**28.5**	**30.31**
